# The relationship between social media interaction quality and loneliness in older adults: the moderating role of perceived social support

**DOI:** 10.3389/fpsyg.2026.1767929

**Published:** 2026-08-07

**Authors:** Jing Han

**Affiliations:** Architecture and Art College, Zhejiang Business Technology Institute, Ningbo, Zhejiang, China

**Keywords:** interaction quality, loneliness, older adults, perceived social support, quantile regression, Social Compensation Theory, social media

## Abstract

**Objective:**

Moving beyond the traditional focus on social media frequency, this study examines the cross-sectional association between perceived social media interaction quality and loneliness among older adults, and the moderating role of perceived social support. This study seeks to provide associational evidence to inform future research.

**Methods:**

A cross-sectional survey recruited 512 social media-using Chinese older adults aged ≥60. Data were collected using an adapted Social Media Interaction Quality item set, an adapted 6-item UCLA-based loneliness measure, and an abbreviated perceived social support scale (MSPSS). Analyses employed Latent Moderated Structural Equation Modeling and Quantile Regression.

**Results:**

Adjusting for demographics, social media interaction quality was negatively associated with loneliness (*β* = −0.32, *p* < 0.001); Perceived social support positive moderating this relationship (*β* = 0.12, *p* < 0.001), the negative was stronger among older adults with lower perceived social support. Quantile regression indicated the negative association remained broadly stable across the loneliness distribution though modestly attenuated at the highest quantile (Q90: −0.22 vs. Q10: −0.34).

**Conclusion:**

The psychological correlates of older adults’ social media use might depend more on interaction quality than quantity Given the cross-sectional design, these findings provide associational evidence—not causal proof—that high-quality online interaction relates to lower loneliness. Future longitudinal research is needed to test interventions among socially vulnerable groups.

## 1 Introduction

As we advance into the 21st century, population aging has become an irreversible global trend. The World Health Organization (WHO) projects that by 2050, the global population aged 60 and over will reach 2.1 billion (World Health Organization, 2025). This process is particularly rapid in China. Accompanied by the nuclearization of family structures, a surge in “empty-nest” seniors, and community atomization driven by urbanization, the traditional social connections of older adults are gradually fracturing, making “loneliness” a prevalent public health crisis (Holt-Lunstad, 2024; Donovan and Blazer, 2020). A vast body of cutting-edge research indicates that chronic loneliness is not merely a subjective painful experience but also a strong predictor of multiple adverse health outcomes. It is closely associated with a 29% increased risk of cardiovascular disease mortality, accelerated cognitive decline, a doubled risk of developing Alzheimer’s disease, and even an increase in all-cause mortality (Holt-Lunstad et al., 2015). Consequently, finding low-cost, accessible, and effective interventions to help older adults rebuild social connections has become an urgent issue in the field of Healthy Aging (Ruiz-Figueroa et al., 2025).

Simultaneously, the rapid development of digital technology has reshaped the landscape of interpersonal communication, profoundly altering the lives of the older population. On one hand, digital technology appears to offer new opportunities to alleviate loneliness. For many older adults with mobility issues or geographic isolation, social media platforms like WeChat and Douyin (TikTok) transcend time and space, providing a window to maintain intergenerational family ties, expand social networks, and participate in public life (Tripathi et al., 2025). On the other hand, the debate over whether technology can genuinely cure loneliness has never ceased. Academic research on the relationship between social media and loneliness in older adults has long been characterized by two opposing viewpoints. Proponents of the “Social Augmentation Hypothesis” argue that online interactions supplement and enhance offline relationships, thereby reducing loneliness (Shaw and Gant, 2002; Wei et al., 2025). Conversely, studies supporting the “Social Displacement Hypothesis” contend that excessive immersion in the online virtual world crowds out valuable real-world face-to-face communication, thus exacerbating social isolation and psychological alienation (Kraut et al., 1998; Roberts et al., 2024).

This study posits that the root of these contradictions lies in the fact that past research has often focused on the crude metric of “quantity of use,” such as duration of use, login frequency, or time spent online, following the “Digital Access Divide,” while neglecting the more critical “Quality of Use Divide” (Godard and Holtzman, 2024; Lei et al., 2024). Use quantity is theoretically ambiguous: longer time spent on social media may reflect active relational maintenance, but it may also indicate passive browsing, compensatory coping, or rumination among lonely users. Therefore, quantity-based indicators may mix qualitatively different forms of engagement and partly explain the inconsistent findings in previous studies. For older adults yearning for emotional comfort, “time spent online” does not equate to “quality of connection.” Passively browsing a newsfeed for extended periods may induce upward social comparison and lead to psychological distress. In contrast, active communication characterized by emotional depth, timely responses, and mutual care—which we define as the core construct of “Interaction Quality”—is key to building psychological safety and a sense of belonging (Nguyen et al., 2025; Maclean, 2025). Nevertheless, the relationship between interaction quality and loneliness may also be shaped by self-selection, offline social skills, personality traits, digital literacy, and platform affordances. Thus, high-quality interaction may be associated with lower loneliness, but loneliness may also influence how older adults initiate, maintain, or perceive online interactions. Therefore, this study aims to re-examine the psychological correlates of digital technology from the dimension of “quality.” Based on this, we propose our first hypothesis:

*H1*: Social Media Interaction Quality is significantly and negatively associated with loneliness in older adults.

Furthermore, if high-quality online interactions are associated with lower loneliness, are these associations equally distributed among all older adults, or do they depend on specific population characteristics? The “Social Compensation Theory” provides a powerful theoretical lens. Social Compensation Theory, as evidenced by empirical work (Valkenburg and Peter, 2007), posits that the effects of media are not uniform. It primarily benefits individuals who lack social resources or skills in real life, compensating for their real-world deficiencies (Ma et al., 2023). According to this reasoning, for older adults with abundant support from friends and family offline, online interaction may merely supplement existing support. However, for those who are living alone, widowed, or have children living far away, high-quality online interaction may play a crucial supplementary or compensatory role, becoming a vital component of their social support network (Hussain et al., 2023). In the present cross-sectional study, however, “compensation” is used as a theoretical interpretation of an observed moderation pattern rather than as evidence of a causal compensatory relationship. Specifically, if the interaction term between interaction quality and perceived social support is positive while the main coefficient for interaction quality is negative, this would indicate that the negative association between interaction quality and loneliness becomes weaker as perceived social support increases, or equivalently, stronger among older adults with lower perceived social support. We therefore expect perceived social support to moderate the association between interaction quality and loneliness. Accordingly, we propose our second hypothesis:

*H2*: Perceived social support moderates the relationship between social media interaction quality and loneliness. Specifically, the negative association between high-quality online interaction and loneliness is stronger for older adults with low levels of perceived social support compared to those with high levels.

Moreover, traditional linear regression analysis only focuses on the average association of variables, which may have limitations in public health practice as it masks the heterogeneous associations of a predictor across groups with varying degrees of distress (Vidal et al., 2020). We are most concerned with the high-risk individuals at the tail end of the loneliness distribution (i.e., the loneliest). To this end, this study employs quantile regression to map the differential associations between interaction quality and loneliness across different loneliness levels, aiming to answer the precision public health question: “For which loneliness-level groups is the association between interaction quality and loneliness more pronounced?” Given the cross-sectional design, the quantile regression results are interpreted as evidence of association heterogeneity rather than evidence of differential intervention efficacy.

## 2 Materials and methods

### 2.1 Study design, participants, and procedure

This study employed a cross-sectional survey design, with data collected between March and May 2025. To enhance sample heterogeneity, a hybrid online and offline convenience sampling strategy was adopted. Offline, we conducted face-to-face assisted questionnaire completion at community activity centers, targeting older adults who had recent experience using smartphone-based social applications but were less proficient in independently completing online questionnaires. Online, questionnaires were distributed through online social groups associated with a university for the elderly. Specifically, the final sample included 89 participants from the offline-assisted stream and 423 participants from the online stream.

Inclusion criteria were: (1) age ≥60 years; (2) experience using smartphone social applications (e.g., WeChat, Douyin) within the past month; and (3) being conscious and possessing basic comprehension and expression abilities. Because recent social media use was an inclusion criterion, participants in the offline-assisted stream were older social media users who required assistance in completing the questionnaire, rather than non-users of social media. Thus, the sample represents older adults with at least minimal recent experience of smartphone-based social applications. An initial 630 questionnaires were collected. After a data cleaning process—which involved removing samples that did not meet the age criteria, exhibited linear response patterns, or had more than 15% missing data on key variables—a final valid sample of 512 was retained, yielding an effective response rate of 81.3%.

### 2.2 Measures

The measures used in this study included both established instruments and adapted item sets. Because several measures were adapted for the present older-adult social media context, their psychometric properties were evaluated in the current sample. Unless otherwise specified, a 5-point Likert scale was used. The full items for all scales and the operational definitions of control variables are provided in the Supplementary material.

Social Media Interaction Quality (IQ) was measured using a five-item study-specific item set developed for the present study and informed by prior work on online supportive interaction (Oh et al., 2014). This item set assesses the perceived responsiveness, emotional support, and reciprocity in older adults’ social media interactions. Recent research has also emphasized the importance of distinguishing interaction quality from quantity (Grotto and Buja, 2025). An example item is: “When I message or comment to family/friends online, I usually get a timely response.” The items focus on experienced two-way communication on social media, including timely responses, emotional authenticity, perceived care, reciprocity, and overall communication satisfaction. In the current study, this item set demonstrated acceptable internal consistency for an exploratory measure (Cronbach’s *α* = 0.63).

Perceived Social Support (PSS) was measured using an abbreviated six-item measure based on the family and friend support domains of the Multidimensional Scale of Perceived Social Support (MSPSS) framework developed by Zimet et al. (1988). This measure assesses perceived support resources from family members and friends, which are common everyday support sources for older adults (Lee et al., 2022; Drageset, 2021). The significant-other domain was not included because this study focused on family and peer support in older adults’ daily social networks. An example item is: “I can share my joys and sorrows with my friends.” In our study, this abbreviated measure showed excellent internal consistency (Cronbach’s *α* = 0.77).

Loneliness was measured using an adapted six-item UCLA-based loneliness measure. The items were selected and adapted from the broader UCLA loneliness scale tradition (Russell et al., 1980) and the short-form literature (Neto, 2014) to assess perceived social isolation and relational disconnection among older adults. This measure was used as an adapted six-item item set rather than as the standard ULS-6, because the items were adjusted to fit the present survey context and were phrased in the same direction to facilitate understanding among older participants. An example item is: “I often feel left out.” The scale uses a 4-point scoring system (1 = Never, 4 = Always), with higher scores indicating greater loneliness. In this study, the scale demonstrated good internal consistency (Cronbach’s *α* = 0.77).

#### 2.2.1 Control variables

This study collected data on age, gender, education level, living status, self-rated health, and average daily social media usage time as control variables. The detailed measurement methods and coding schemes for these variables are provided in Table B1 of the Supplementary material. Recruitment stream was also recorded to describe the sample composition and compare key characteristics between the offline-assisted and online survey streams.

### 2.3 Data analysis

This study followed a multi-step statistical analysis procedure. First, SPSS 26.0 was used for descriptive statistics, common method bias testing (ULMC method), difference testing, and comparisons between recruitment streams. Second, Mplus 8.3 was used for confirmatory factor analysis (CFA) to assess the reliability and validity of the latent constructs. The CFA results included standardized factor loadings, composite reliability (CR), average variance extracted (AVE), and discriminant validity. The core analysis employed Latent Moderated Structural Equation Modeling (LMS) to test main and moderation associations while accounting for measurement error. For models without the latent interaction term, standard global fit indices were reported. For the LMS model containing the latent interaction term, model evaluation was based on information criteria, explained variance, and the statistical significance of the interaction term, because conventional global fit indices are not directly available for latent interaction models estimated using LMS. Finally, to explore heterogeneous associations, Stata 17.0 was used to perform Quantile Regression at five quantiles: 0.10, 0.25, 0.50, 0.75, and 0.90. All quantile regression models included the same control variables as the main model, and the results were interpreted as descriptive evidence of association heterogeneity. All tests were two-tailed, and a *p*-value < 0.05 was considered statistically significant.

## 3 Results

### 3.1 Common method bias and preliminary analysis

As all data were self-reported, the Unmeasured Latent Method Construct (ULMC) approach was first used to test for common method bias (CMB). As shown in Table 1, after introducing a method factor to the three-factor CFA model, the model fit indices did not show substantial improvement (ΔCFI = 0.006, ΔTLI = 0.004, ΔRMSEA = −0.003), indicating that there was no severe common method bias in this study’s data. Although the chi-square value decreased after adding the method factor, the changes in approximate fit indices were very small. Therefore, the assessment of common method bias was based primarily on changes in CFI, TLI, and RMSEA rather than on the chi-square difference alone.

**Table 1:** Results of common method bias test.

Model setup	χ^2^	df	CFI	TLI	RMSEA	Comparison
M1: Baseline Theoretical Model	144.7	116	0.983	0.980	0.022	–
M2: Model with Method Factor	114.2	96	0.989	0.984	0.019	M2 vs. M1
Difference (*Δ*)	30.5	20	0.006	0.004	−0.003	Approximate fit improvement was negligible

The chi-square difference is reported descriptively. Because chi-square difference testing is sensitive to sample size and may require estimator-specific correction, the evaluation of common method bias focused on changes in approximate fit indices. The changes in CFI, TLI, and RMSEA were very small, suggesting that common method bias was unlikely to substantially distort the substantive findings.

The demographic characteristics of the sample and descriptive statistics for key variables are presented in Table 2. The 512 participants included in the final analysis had an average age of 67.90 years (*SD* = 5.35), ranging from 60 to 85 years. Females were slightly in the majority (*n* = 280, 54.7%). Regarding living arrangements, the vast majority of participants lived with family (*n* = 377, 73.6%), but nearly one-fifth (*n* = 100, 19.5%) were living alone, with a small minority (*n* = 35, 6.8%) residing in nursing homes. Over half of the participants (*n* = 291, 56.8%) rated their health as “good” or “very good.” These sample characteristics provide a descriptive profile of older adult social media users in the present sample. Because this study used convenience sampling and required recent experience with smartphone-based social applications, the findings should be interpreted within this sampling context rather than generalized to all older adults.

**Table 2:** Analysis of key variable differences across demographic groups.

Variable	Group	*N*	Interaction quality (Mean ± SD)	Social support (Mean ± SD)	Loneliness (Mean ± SD)	Test statistic
Gender	Male	232	3.44 ± 0.79	3.65 ± 0.77	2.35 ± 0.69	*t* = −0.10
	Female	280	3.39 ± 0.72	3.63 ± 0.78	2.36 ± 0.66	
Living status	Living alone	100	3.27 ± 0.78	3.33 ± 0.73	2.54 ± 0.72	*F* = 5.06**
	With family	377	3.44 ± 0.74	3.73 ± 0.77	2.30 ± 0.66	
	Nursing home	35	3.52 ± 0.78	3.53 ± 0.76	2.42 ± 0.61	
Self-rated health	Poor/Fair	221	3.42 ± 0.73	3.59 ± 0.79	2.45 ± 0.70	*t* = 3.37***
	Good/Very good	291	3.41 ± 0.77	3.68 ± 0.76	2.26 ± 0.64	

**p < 0.01, ***p < 0.001. Differences for gender and self-rated health on loneliness were tested using independent-samples *t*-tests; differences for living status on loneliness were tested using one-way ANOVA.

Analysis of sample characteristics (Table 2) showed that the loneliness score of older adults living alone (*M* = 2.54, *SD* = 0.72) was significantly higher than that of those living with family (*M* = 2.30, *SD* = 0.66). The main association of living status with loneliness was significant in a one-way ANOVA (*F*(2, 509) = 5.06, *p* = 0.007). This provides an empirical backdrop for the subsequent examination of the moderation pattern. A post-hoc LSD test further revealed that the loneliness scores of those living alone were significantly higher than those living with family (*p* = 0.002), while there was no significant difference in loneliness levels between older adults in nursing homes (*M* = 2.42, *SD* = 0.61) and the other two groups (*ps* > 0.05). Furthermore, older adults with better self-rated health also reported significantly lower levels of loneliness (*t* = 3.37, *p* < 0.001). However, no significant differences in loneliness were observed between male and female participants in this sample (*t* = −0.10, *p* = 0.921).

In addition, recruitment-stream comparisons were conducted to describe whether the offline-assisted and online survey streams differed in demographic and key study variables. The final sample included 89 participants from the offline-assisted stream and 423 participants from the online stream. As shown in Table 3, no statistically significant differences were observed between the two streams in age, gender composition, living-alone status, interaction quality, perceived social support, loneliness, or daily social media usage time.

**Table 3:** Characteristics of participants by recruitment stream.

Variable	Offline-assisted stream	Online stream	Test statistic
*N*	89	423	–
Age, Mean ± SD	68.06 ± 5.56	67.87 ± 5.32	*t* = 0.29, *p* = 0.769
Female, *n* (%)	43 (48.3%)	237 (56.0%)	*χ*^2^ = 1.47, *p* = 0.226
Living alone, *n* (%)	16 (18.0%)	84 (19.9%)	*χ*^2^ = 0.07, *p* = 0.795
Interaction quality, Mean ± SD	3.45 ± 0.75	3.41 ± 0.76	*t* = 0.46, *p* = 0.649
Perceived social support, Mean ± SD	3.57 ± 0.80	3.65 ± 0.77	*t* = −0.89, *p* = 0.375
Loneliness, Mean ± SD	2.35 ± 0.64	2.36 ± 0.68	*t* = −0.09, *p* = 0.932
Daily social media use time, Mean ± SD	2.10 ± 1.11	2.10 ± 1.02	*t* = 0.01, *p* = 0.996

Differences between recruitment streams were tested using independent-samples *t*-tests for continuous variables and chi-square tests for categorical variables.

The descriptive statistics and correlation matrix for all variables are presented in Table 4. Social media interaction quality was significantly negatively correlated with loneliness (*r* = −0.41, *p* < 0.001), and perceived social support was also significantly negatively correlated with loneliness (*r* = −0.44, *p* < 0.001). No severe multicollinearity issues were found among the variables.

**Table 4:** Descriptive statistics and correlation matrix of variables.

Variable	Mean	SD	α	1 (Age)	2 (Health)	3 (IQ)	4 (PSS)	5 (Loneliness)
1. Age	67.90	5.35	-	1				
2. Health	2.67	0.87	-	0.03	1			
3. Interaction quality (IQ)	3.41	0.75	0.63	0.03	0.05	1		
4. Social support (PSS)	3.64	0.77	0.77	−0.01	0.00	0.20***	1	
5. Loneliness (UCLA)	2.35	0.67	0.77	0.00	−0.18***	−0.41***	−0.44***	1

***p < 0.001.

### 3.2 Measurement model and construct validity

Before testing the structural model, a three-factor confirmatory factor analysis was conducted to examine the measurement structure of Social Media Interaction Quality, Perceived Social Support, and Loneliness. The measurement model yielded an excellent fit to the data (*χ*^2^/*df* = 1.25, CFI = 0.983, TLI = 0.980, RMSEA = 0.022). Standardized factor loadings, composite reliability, average variance extracted, and discriminant validity were examined because several measures were adapted for the present study. The CFA results supported the use of the three latent constructs in the subsequent structural analyses, although the adapted nature of the item sets should be considered when interpreting the findings. Detailed measurement model parameter estimates, including standardized factor loadings, CR, and AVE, are provided in Table 5.

**Table 5:** Results of confirmatory factor analysis for measurement model.

Construct	Items	Standardized loading	Composite reliability (CR)	Average variance extracted (AVE)
Interaction quality (IQ)	IQ1	0.469***	0.630	0.255
	IQ2	0.519***		
	IQ3	0.549***		
	IQ4	0.446***		
	IQ5	0.535***		
Perceived social support (PSS)	PSS1	0.598***	0.768	0.356
	PSS2	0.574***		
	PSS3	0.573***		
	PSS4	0.637***		
	PSS5	0.575***		
	PSS6	0.620***		
Loneliness (UCLA)	UCLA1	0.612***	0.765	0.353
	UCLA2	0.590***		
	UCLA3	0.626***		
	UCLA4	0.582***		
	UCLA5	0.604***		
	UCLA6	0.547***		

***p < 0.001. All factor loadings were statistically significant. AVE values for all three constructs were below 0.50, indicating limited convergent validity by conventional psychometric standards; these estimates should be interpreted with appropriate caution. Discriminant validity was supported: inter-construct correlations (|*r*| ≤ 0.44) were each smaller than the square root of the corresponding AVE (√AVE: IQ = 0.505, PSS = 0.597, UCLA = 0.594), consistent with the Fornell–Larcker criterion.

### 3.3 Main associations and moderation analysis

To test the necessity of the moderation term, a nested model comparison was performed (Table 6). The results showed that Model 2, which included the latent interaction term, had the best relative model performance based on information criteria (AIC = 1252.0, *R*^2^ = 0.35). Compared to Model 1, which only included main associations, Model 2 showed a lower AIC value and higher explained variance, supporting the inclusion of the moderation term.

**Table 6:** Nested comparison analysis.

Model	Predictors included	AIC	*R*^2^ (Loneliness)	Conclusion
Model 0	Control variables only	1437.6	0.05	Baseline comparison model
Model 1	Main effects added (IQ, PSS)	1261.0	0.33	Improved relative performance
Model 2	Latent interaction term added (IQ × PSS)	1252.0	0.35	Best relative performance among compared models

Models were evaluated using Akaike Information Criterion (AIC) and explained variance (*R*^2^). Lower AIC values indicate better relative model performance. Compared to Model 1, Model 2 showed a decrease of 9.0 in AIC and a structural increase in explained variance, supporting the inclusion of the moderation term.

**Figure 1:**
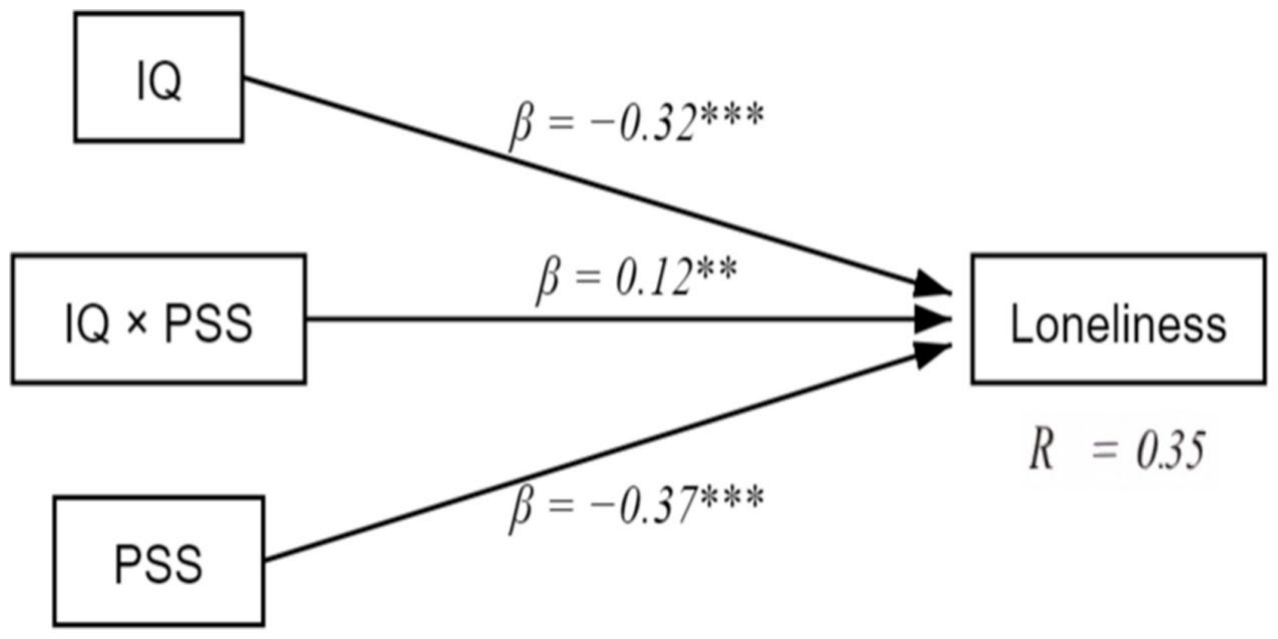
Structural equation model with standardized path coefficients. Interaction quality (IQ) and perceived social support (PSS) predicting loneliness, controlling for age, gender, education level, self-rated health, living status, and daily social media use time. *R*^2^ = 0.35. ***p < 0.001.

Based on the optimal Model 2, the specific path coefficients are shown in Table 7 and Figure 1. After controlling for variables such as age, gender, health status, living status, and usage time, interaction quality had a significant negative association with loneliness (*β* = −0.32, *p* < 0.001), supporting H1. Perceived social support also had a significant negative association with loneliness (*β* = −0.37, *p* < 0.001). The crucial interaction term “Interaction Quality × Social Support” was significantly positive (*β* = 0.12, *p* < 0.001), supporting H2. Because the main association between interaction quality and loneliness was negative, the positive interaction coefficient indicates that this negative association became weaker as perceived social support increased. Equivalently, the association between interaction quality and loneliness was stronger among older adults with lower perceived social support.

**Table 7:** Path coefficients of the structural model.

Path	Standardized Coeff. (*β*)	*t*-value	*p*-value	Result
Control variables				
Self-rated health → Loneliness	−0.16	−4.46	<0.001	Significant
Living alone → Loneliness	0.07	0.78	0.436	Not significant
Core paths				
H1: Interaction quality → Loneliness	−0.32	−8.58	<0.001	Supported
Social support → Loneliness	−0.37	−9.75	<0.001	Significant
H2: IQ × PSS (Interaction) → Loneliness	0.12	3.31	<0.001	Supported

Age, gender, education level, and daily social media use time were also included as control variables in the structural model but are not displayed in the table for brevity; their inclusion did not alter the substantive findings.

Notably, among the control variables, self-rated health (*β* = −0.16, *p* < 0.001) maintained a significant negative association with loneliness in the full model. However, the effect of living alone was no longer statistically significant in the multivariate model (*β* = 0.07, *p* = 0.436), possibly because the variance was largely absorbed by perceived social support and health status. The entire model explained 35% of the total variance in loneliness (*R*^2^ = 0.35).

**Figure 2:**
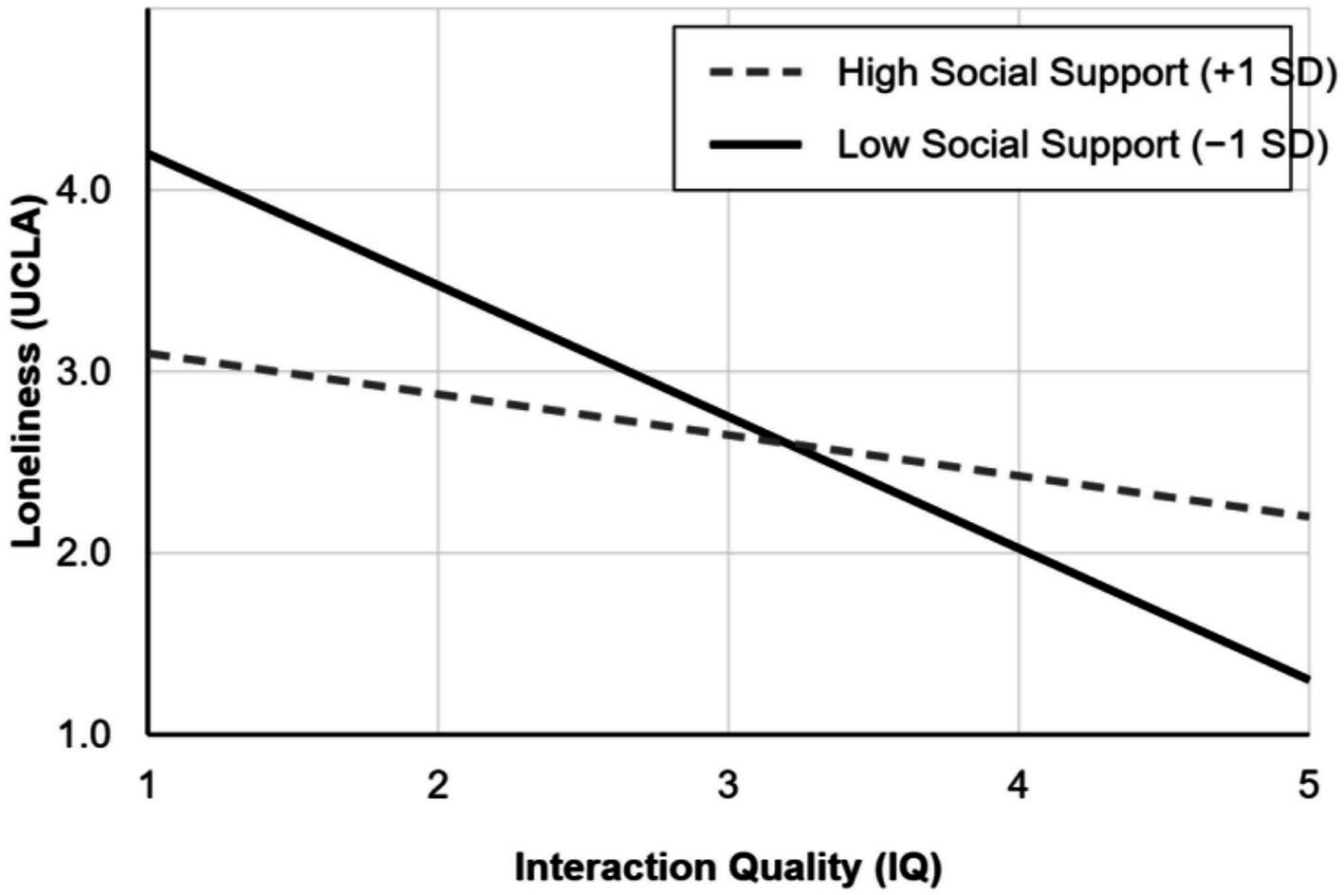
Simple slopes of the interaction between social media interaction quality and perceived social support on loneliness. Solid line = low social support (−1 SD); Dashed line = high social support (+1 SD).

To clearly interpret the pattern of the moderation association, we conducted a simple slope analysis and plotted the interaction pattern (Figure 2). The results show that for the low social support group (−1 SD), the negative relationship between interaction quality and loneliness was stronger (*simple slope* = −0.44, *p* < 0.001). In contrast, for the high social support group (+1 SD), this negative relationship became relatively flatter but remained significant (*simple slope* = −0.20, *p* < 0.001). This pattern is consistent with the social compensation perspective, but it should be interpreted as an observed moderation pattern rather than as evidence of a causal compensatory relationship.

### 3.4 Heterogeneity analysis of associations

**Figure 3:**
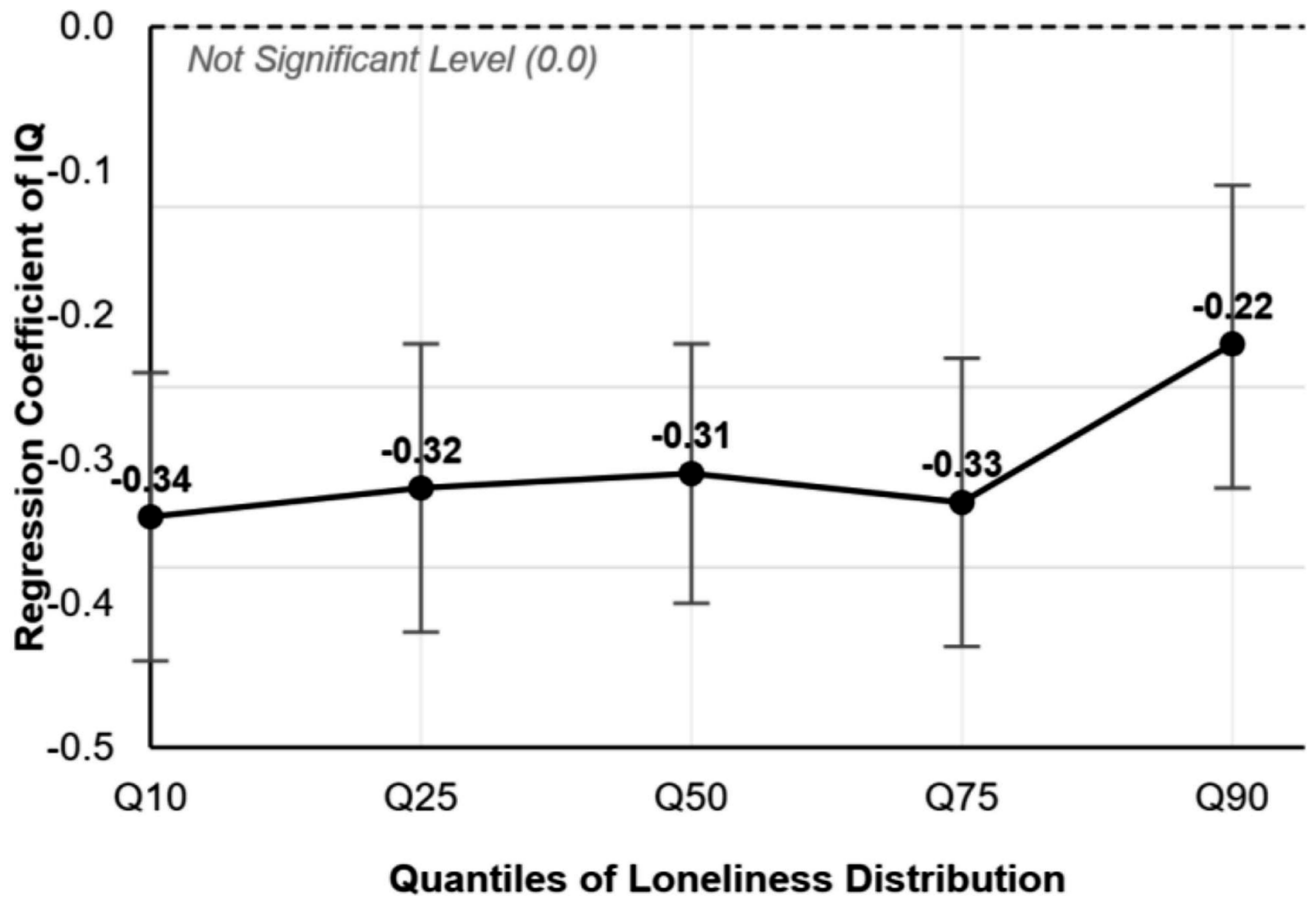
Quantile regression coefficients of interaction quality predicting loneliness across five quantiles (Q10–Q90). Dots = point estimates; vertical bars = 95% confidence intervals. All *p*s < 0.001.

To further investigate whether the association of interaction quality is heterogeneous across the loneliness distribution—and importantly, to address potential concerns regarding floor effects at the lower end of the measurement scale—we conducted quantile regression using the cleaned dataset. The detailed results are presented in Table 8 and Figure 3.

**Table 8:** Quantile regression results: association differences at various loneliness levels.

Quantile	Quantile regression coefficient	95% Confidence interval (CI)	*p*-value
Q10 (Very Low)	−0.34	[−0.44, −0.24]	<0.001
Q25 (Low)	−0.32	[−0.42, −0.22]	<0.001
Q50 (Medium)	−0.31	[−0.40, −0.22]	<0.001
Q75 (High)	−0.33	[−0.43, −0.23]	<0.001
Q90 (Very High)	−0.22	[−0.32, −0.11]	<0.001

The coefficients indicate conditional associations between interaction quality and loneliness at different quantiles of the loneliness distribution. These results should not be interpreted as causal effects.

Figure 3 visually demonstrates that the negative coefficient of interaction quality was broadly stable across the entire loneliness spectrum. In contrast to unadjusted models that might suggest a steep monotonic increasing trend (which could be influenced by measurement floor effects at the lower end of the scale), our fully adjusted quantile regression shows that the negative association between interaction quality and loneliness was consistently observed across groups, from those experiencing very low loneliness (Q10: Coeff. = −0.34, *p* < 0.001) to those reporting very high loneliness levels (Q90: Coeff. = −0.22, *p* < 0.001).

Overall, the quantile regression results suggest that the negative association between interaction quality and loneliness was broadly consistent across quantiles. While floor effects at the lower end of the distribution cannot be fully ruled out, the pattern does not appear to be driven solely by measurement artifacts. These results are consistent with the view that the negative association of interaction quality with loneliness is not confined to those experiencing extreme levels of loneliness, but rather appears across older adults with a range of loneliness profiles.

## 4 Discussion

Through a large-sample survey and two complementary statistical methods—latent moderated structural equation modeling and quantile regression—this study systematically explored the relationship between social media interaction quality and loneliness in Chinese older adults. The results provide a quality-oriented perspective for understanding digital interaction in later life and offer empirical implications for future research on loneliness and social connectedness among older adults.

### 4.1 Interpretation of key findings

#### 4.1.1 The primacy of quality over quantity in digital interaction

The primary finding of this study is that, after controlling for usage time, interaction quality has an independent and robust negative association with loneliness. This finding helps clarify the long-standing contradictory conclusions in academia regarding the relationship between social media use and well-being. It aligns with some younger or general adult populations, which found that active, meaningful interaction, rather than passive use, is positively associated with well-being (Mao et al., 2023), and suggests that a similar quality-oriented perspective may also be relevant for older adults. More importantly, this finding nuances the pessimistic view of some studies that social media use inevitably leads to social displacement (Roberts et al., 2024). It reveals a potential limitation of such research—namely, the failure to effectively distinguish between the physical state of being “online” and the psychological state of being “connected,” conflating the essential distinction between “quantity of use” and “effective connection” (Godard and Holtzman, 2024). This study suggests that for older adults, social media may be understood as a double-edged context; its relationship with loneliness may vary depending on whether users experience responsive and emotionally meaningful interaction (Kim and Lee, 2023). In this sense, the findings support a shift from asking how long older adults use social media to examining what kinds of interactions they experience during use.

#### 4.1.2 Social compensation pattern: digital support for older adults with lower offline support

One of the findings contributions of this study is the results provide associational evidence consistent with the social compensation perspective in the context of digital inclusion for older adults. The moderation analysis showed that the negative association between high-quality online interaction and loneliness was more pronounced among older adults with insufficient real-world social support. This aligns with the empirical finding from our descriptive statistics that “older adults living alone experience greater loneliness.”

This result contrasts with phenomena observed in some studies where individuals rich in real-world social connections benefit more online (Burke et al., 2010), revealing that different populations may follow different logics when using digital technology. For the specific group of older adults, the primary motivation for using social media is often to maintain existing emotional connections rather than to expand new relationships (Liu and Yeo, 2024). Therefore, when real-world support networks are compromised, high-quality online interaction may serve as an important supplementary relational resource. This highlights the potential of digital technology to support social connection when offline support is relatively limited (Wu and Chiou, 2023).

It should be emphasized that the term “compensation” is used here as a theoretical interpretation of the observed moderation pattern rather than as evidence of a causal compensatory process. The present cross-sectional data show that interaction quality is more strongly related to loneliness among older adults with lower perceived social support, but they do not establish that improving online interaction quality would necessarily reduce loneliness over time.

#### 4.1.3 Association heterogeneity across the loneliness distribution

The heterogeneous associations revealed by quantile regression provide useful distributional information beyond average associations estimated by traditional OLS regression. The results showed that the negative association between interaction quality and loneliness was observed across all examined quantiles and was broadly stable, although the coefficient was modestly attenuated at the highest loneliness quantile (Q90 = −0.22 vs. Q10 = −0.34). Thus, the findings do not indicate that the association was strongest among the loneliest participants; rather, they suggest that interaction quality remained negatively associated with loneliness across a wide range of loneliness profiles, including those reporting higher levels of loneliness. This distributional perspective is consistent with recent calls to move beyond aggregate indicators of social media use and to examine the heterogeneity of psychosocial outcomes in older adults’ digital social experiences (Lei et al., 2024).

From a methodological viewpoint, it is important to acknowledge that this pattern might also be partially influenced by statistical floor effects. Because individuals at the lowest quantiles, such as Q10, already report relatively low loneliness scores on the measurement scale, the mathematical room for further decreases in loneliness is inherently constrained. Therefore, the observed quantile pattern should be interpreted cautiously as descriptive evidence of association heterogeneity rather than as direct evidence of differential intervention efficacy. Nevertheless, by showing that the negative association between interaction quality and loneliness appears across multiple points of the loneliness distribution, the quantile regression results provide useful information for future research on digital responses to loneliness among older adults (Ruiz-Figueroa et al., 2025).

### 4.2 Implications of the study

#### 4.2.1 Theoretical implications

This study makes three main theoretical contributions. First, it not only offers associational evidence consistent with the Social Compensation Theory in the older adult population but also extends and refines it by revealing that the strength of the observed moderation pattern depends on the degree of deficit in real-world support. Second, through quantile regression, this study adds a distributional perspective to this framework, showing that the negative association between interaction quality and loneliness may vary descriptively across the loneliness distribution rather than being uniform across all older adults. Because the coefficients essentially remained flat from Q10 to Q75 with only a modest attenuation at Q90, and without a formal inter-quantile equality test, this broad stability—rather than a distinct heterogeneity pattern—offers a useful lens for future digital health research. Finally, this study adds to the relevant research field by reinforcing a paradigm shift from focusing on the “first digital divide” (access) to the “second and third digital divides” (skills of use and ultimate outcomes) (Godard and Holtzman, 2024; Grotto and Buja, 2025).

#### 4.2.2 Practical and policy implications

Based on these associational findings, this study suggests several tentative practice and policy implications for supporting social connectedness among older adults. At the public policy level, governments and social organizations may consider incorporating “digital interaction literacy” into elderly education and community health service systems. Relevant training should not stop at teaching how to use software but should focus on three core skill modules: (1) techniques for initiating meaningful conversations (e.g., sharing life moments rather than just forwarding articles); (2) strategies for identifying and handling negative emotions online (e.g., how to cope with information anxiety or disputes in family group chats); and (3) methods for establishing high-quality online ‘micro-communities’ (e.g., forming small groups based on common hobbies) (Kim and Lee, 2023). At the community service level, community workers and family doctors could consider using validated brief scales like the MSPSS or ULS-6 to conduct preliminary screenings of older adults in their jurisdictions, focusing on identifying individuals who live alone or have high levels of loneliness. They may then provide targeted online interaction support, such as organizing online interest groups or pairing them with “digital buddies,” to respond to the observed pattern that interaction quality appears more closely related to loneliness among socially vulnerable older adults (Tripathi et al., 2025). At the product design level, technology companies, when designing age-friendly apps, should go beyond enlarging fonts and simplifying workflows to incorporate “emotional design” (Ma et al., 2023). For example, they could optimize the usability of video calls, add “strong connection” features like one-click voice-to-text, or even have the system automatically send a comforting message like, “Your family member may be busy right now; please take a short break,” when a message goes unanswered for a long time, to proactively manage the negative expectations of older users. Finally, at the family level, this study reminds the younger generation that high-quality online companionship for their parents—a patient video call, a timely voice reply—is an extremely important form of “Digital Filial Piety” in the new era, with emotional value that cannot be replaced by material support alone (Liu and Yeo, 2024).

## 5 Limitations and prospects

Although this study yielded meaningful conclusions, several limitations remain. First, the cross-sectional design, while able to reveal correlations between variables, cannot be used to infer strict causality. Future research should employ longitudinal study designs (Roberts et al., 2024) or diary studies to track the dynamic changes of variables over time to more accurately capture temporal ordering and possible directional relationships. For example, although higher interaction quality was associated with lower loneliness in this study, it is also possible that older adults with lower loneliness, stronger offline social skills, or greater digital confidence are more likely to perceive online interactions as responsive and emotionally meaningful. This concern is underscored by longitudinal evidence: Roberts et al. (2024), drawing on a 9-year dataset, found that passive social media use predicted increased loneliness over time, while active use showed a more nuanced pattern. The present study cannot address such temporal ordering, though the distinction between quality-oriented active interaction and passive consumption is broadly consistent with that longitudinal framework.

Second, the use of convenience sampling may result in a sample that does not fully represent all elderly internet users in China, particularly the “invisible elderly” who are completely disconnected from digital society, which limits the external validity of the research findings to some extent. Because recent experience with smartphone-based social applications was an inclusion criterion, the sample should be understood as older adults with at least minimal recent social media use rather than the broader population of all older adults.

Third, all variables were obtained through self-report measures, which may be subject to social desirability bias. Although the common method bias test suggested that common method variance was unlikely to seriously distort the results, self-report data still cannot fully capture actual online interaction behaviors, such as message frequency, response latency, communication initiations, or the emotional tone of conversations.

Fourth, several measures were adapted for the present older-adult social media context. Although the CFA results supported the overall three-factor structure, two psychometric indicators warrant caution: the Cronbach’s *α* for interaction quality (α = 0.63) fell marginally below the conventional threshold of 0.70—though values above 0.60 are generally considered acceptable in exploratory measurement contexts (Nunnally, 1978)—and AVE values for all three constructs (IQ = 0.255, PSS = 0.356, UCLA = 0.353) fell below the 0.50 threshold recommended by Fornell and Larcker (1981), indicating limited convergent validity by conventional standards. Specifically, the lowest AVE was observed for the interaction quality construct (AVE = 0.255), meaning the latent IQ factor captures roughly a quarter of its indicators’ variance. Because of this measurement limitation, our substantive claims regarding the association between interaction quality and loneliness should be interpreted with appropriate caution. While the current IQ measure shows promise in capturing the subjective nuances of older adults’ digital communication, it is still in need of further psychometric development. Future research should refine and validate these measures in larger, more diverse samples.

Fifth, the quantile regression results provide descriptive evidence of association heterogeneity across different loneliness levels, but they should not be interpreted as evidence of differential intervention efficacy. The observed distributional pattern may reflect unobserved individual differences, measurement characteristics, or contextual factors. Future longitudinal or experimental studies are needed to examine whether improving online interaction quality is followed by subsequent changes in loneliness among older adults with different loneliness profiles.

Building on these limitations, future research can be expanded in the following ways. First, researchers could try to use multi-source data, such as incorporating evaluations of the older adults’ social situations from their children or friends, or collaborating with tech companies to obtain objective behavioral data (e.g., frequency of initiating chats, response time) to more objectively measure interaction quality (Lei et al., 2024). Second, future studies could more finely distinguish between different social platforms (e.g., strong-tie WeChat vs. weak-tie Douyin) and different interaction formats (e.g., one-on-one private chats vs. group chats) and explore their differential relationships with loneliness (Godard and Holtzman, 2024; Grotto and Buja, 2025). Third, future research may combine quantitative surveys with qualitative interviews to better understand how older adults define “high-quality” online interaction and how such interaction is embedded in their offline family and community networks.

## 6 Conclusion

This study suggests that in an era where aging and digitalization are deeply intertwined, the key to understanding and addressing loneliness in older adults lies not in the “duration of use” of social media, but in the “quality of interaction.” High-quality online interactions were negatively associated with loneliness, and this association was stronger among older adults with lower perceived social support. In the quantile regression analysis, the negative association appeared across the examined loneliness distribution, with modest attenuation at the highest quantile, rather than being confined to a single loneliness subgroup. Therefore, future public health interventions and social support strategies should move beyond the surface level of digital access and focus on supporting older adults in engaging in high-quality digital interactions, which may be associated with more context-sensitive support for their mental health and social connectedness.

## Data Availability

The original contributions presented in the study are included in the article/Supplementary material, further inquiries can be directed to the corresponding author.
