# Supplementary material for: The relationship between social media interaction quality and loneliness in older adults: the moderating role of perceived social support
Source: Front Psychol. 2026 Aug 7;17:1767929. doi: 10.3389/fpsyg.2026.1767929 (PMC13493543; doi:10.3389/fpsyg.2026.1767929)
Supplement: Supplementary file 1 [file Data_sheet_1.docx]

Supplementary Material

# 1 Supplementary Data

**Appendix A: Measurement Items**

**Table A1. Interaction Quality (IQ)**

| Item Code | English Item (Construct) | Chinese Item (Original used in survey) |
| --- | --- | --- |
| IQ1 | When I message or comment to family/friends online, I usually get a timely response. | 当我在网上给亲友发消息或评论时，通常能得到及时的回应。 |
| IQ2 | I feel that interactions with others on social media are genuine and emotional. | 我觉得在社交媒体上与他人的互动是充满真情实感的。 |
| IQ3 | Through online interactions, I can feel that others care about and value me. | 通过网上的互动，我能感受到对方对我的关心和重视。 |
| IQ4 | My communication on social media is usually mutual, not one-sided. | 我在社交媒体上的交流通常是相互的（有来有往），而不是单方面的。 |
| IQ5 | Overall, I am satisfied with the quality of communication with others on social media. | 总的来说，我对在社交媒体上与他人的沟通质量感到满意。 |

**Table A2. Perceived Social Support (MSPSS)**

| Item Code | English Item | Chinese Item |
| --- | --- | --- |
| PSS1 | My family really tries to help me. | 我的家人真的尽力帮助我。 |
| PSS2 | I get the emotional help and support I need from my family. | 我能从家人那里得到我所需要的情感帮助和支持。 |
| PSS3 | I can talk about my problems with my family. | 我能与家人谈论我的难题。 |
| PSS4 | My friends really try to help me. | 我的朋友真的尽力帮助我。 |
| PSS5 | I can count on my friends when things go wrong. | 当我有困难时，我可以依靠我的朋友。 |
| PSS6 | I can share my joys and sorrows with my friends. | 我能与朋友分享我的快乐和忧愁。 |

**Table A3. Loneliness (ULS-6)**

| Item Code | English Item | Chinese Item |
| --- | --- | --- |
| UCLA1 | I feel that there is no one I can feel close to. | 我感到身边没有可以亲密相处的人。 |
| UCLA2 | I feel that no one really understands me. | 我感到没有人真正了解我。 |
| UCLA3 | I often feel left out. | 我常常感到被冷落。 |
| UCLA4 | I feel isolated from others. | 我感到自己与周围的人格格不入。 |
| UCLA5 | I feel alone. | 我感到只有自己一个人。 |
| UCLA6 | I feel I have no one to turn to when I need help. | 我感到在需要帮助时找不到人^[1]^。 |

**Appendix B: Control Variables and Demographics**

The following table details the measurement and coding of all demographic and control variables used in the analyses.

**Table B1. Demographic and Control Variables**

| Variable (变量) | Measurement and Coding (测量与编码) | Role in Analysis |
| --- | --- | --- |
| Age (年龄) | Measured as a continuous variable in years. 以连续变量（周岁）进行测量。 | Control |
| Gender (性别) | Coded as 0 = Male, 1 = Female. 编码为 0 = 男, 1 = 女。 | Control |
| Education (教育程度) | Measured on a 4-point ordinal scale: 1 = Primary school or below (小学及以下) 2 = Middle school (初中) 3 = High school/Vocational (高中/中专) 4 = College or above (大专/本科及以上) | Control |
| Living Status (居住状况) | Measured with 4 categories, then recoded into a dummy variable for regression analysis: 1 = Living alone, 0 = Others (i.e., with spouse, with children/grandchildren, or in nursing home). 测量时分为4个类别，在回归分析中重新编码为虚拟变量：1 = 独居, 0 = 其他（即与配偶、子女/孙子女同住或住养老机构）。 | Key Control |
| Self-rated Health (自评健康) | Measured on a 5-point scale: 1 = Very poor, 5 = Very good. 采用5点计分：1 = 非常差, 5 = 非常好。 | Control |
| Usage Time (使用时长) | Measured on a 5-point ordinal scale: 1 = < 30 min (少于30分钟) 2 = 30 min - 1 hour (30分钟-1小时) 3 = 1 - 2 hours (1-2小时) 4 = 2 - 3 hours (2-3小时) 5 = > 3 hours (3小时以上) | Control |

1. ¹**Note:** This item was adapted from the original reverse-scored item (“I feel there are people I can turn to”) and rephrased into a direct statement of loneliness to reduce the cognitive load for elderly participants. Consequently, all items in this scale were scored in the same direction.
   ¹**注：** 该题项由原始量表的反向计分题（“我感到在需要帮助时有人可以求助”）改编而来，为降低老年参与者的认知负荷，我们将其修改为同方向的直接陈述。因此，本量表所有题项均按同一方向计分。
